# Association Between Radiomics Signature and Disease-Free Survival in Conventional Papillary Thyroid Carcinoma

**DOI:** 10.1038/s41598-018-37748-4

**Published:** 2019-03-14

**Authors:** Vivian Y. Park, Kyunghwa Han, Eunjung Lee, Eun-Kyung Kim, Hee Jung Moon, Jung Hyun Yoon, Jin Young Kwak

**Affiliations:** 1 Department of Radiology, Severance Hospital, Research Institute of Radiological Science, Yonsei University College of Medicine, Seoul, South Korea; 2 Department of Computational Science and Engineering, Yonsei University, Seoul, South Korea

## Abstract

Patients with papillary thyroid carcinoma (PTC) would benefit from risk stratification tools that can aid in planning personalized treatment and follow-up. The aim of this study was to develop a conventional ultrasound (US)-based radiomics signature to estimate disease-free survival (DFS) in patients with conventional PTC. Imaging features were extracted from the pretreatment US images of 768 patients with conventional PTC who were treated between January 2004 and February 2006. The median follow-up period was 117.3 months, with 85 (11.1%) events. A radiomics signature (Rad-score) was generated by using the least absolute shrinkage and selection operator (LASSO) method in Cox regression. The Rad-score was significantly associated with DFS (hazard ratio [HR], 3.087; *P* < 0.001), independent of clinicopathologic risk factors. A radiomics model which incorporated the Rad-score demonstrated better performance in the estimation of DFS (C-index: 0.777; 95% confidence interval [CI]: 0.735, 0.829) than the clinicopathologic model (C-index: 0.721; 95% CI: 0.675, 0.780). In conclusion, radiomics features from pretreatment US may be potential imaging biomarkers for risk stratification in patients with conventional PTC.

## Introduction

Papillary thyroid carcinoma (PTC) is the most common histologic type of thyroid cancer, and accounts for the majority of increased incidence in thyroid cancer during the last several decades^1,2^. Because of its treatability and relatively favorable survival rate, the perception of thyroid cancer as a “good cancer” has spread among patients and healthcare providers^3,4^. Along with recent guidelines recommending active surveillance rather than treatment in selected patients, some researchers have also suggested expanding this management approach to a larger size range of PTCs^5^. However, a small subset of PTCs shows aggressive clinical behavior, with approximately 9.1–13.3% of patients experiencing recurrence and 1.4–5.2% dying from thyroid cancer^6,7^. Incidence-based mortality for PTC has also increased — increasing 1.1% per year overall and 2.9% per year for distant stage PTC in the United States^2^. Therefore, patients with PTC would largely benefit from preoperative risk stratification tools that can aid in planning appropriate treatment and follow-up.

The field of medical image analysis has grown exponentially in the past decade, fueled by the routine use of digital medical images and developments in methods for quantitative image analysis. Radiomics, a promising field in cancer imaging research, is based on the concept that medical images contain crucial information reflecting underlying pathophysiology, which can be used to support evidence-based clinical decisions^8^. Biomarkers based on quantitative radiomics features have been associated with clinical prognosis and genomic phenotypes across a wide range of cancer types^9–13^. Recently, this approach has also been applied to the analysis of thyroid nodules. Previous studies have reported that sonographic histogram and texture analyses are useful for differentiating benign and malignant thyroid nodules^14–18^. However, to our knowledge, there are no published studies that have investigated whether a radiomics approach can be used to estimate the prognosis of PTC.

Therefore, the aim of this study was to develop a radiomics signature based on thyroid ultrasound (US) images to estimate disease-free survival (DFS) in patients with conventional PTC, and to assess its incremental value to clinical-pathologic risk factors.

## Results

### Clinical Characteristics and Patient Outcomes

Among the 768 patients, 747 (97.3%) underwent total or near-total thyroidectomy, 13 (1.7%) underwent hemithyroidectomy, and 8 (1.0%) underwent hemithyroidectomy with contralateral subtotal thyroidectomy. Table 1 shows the clinicopathologic features of the 768 patients. The median follow-up period was 117.3 months (range, 36.3–154.23 months). At the last follow-up, 85 patients (11.1%) experienced recurrent or persistent disease, with 56 (7.3%) experiencing recurrence and 29 (3.8%) experiencing persistent disease.

**Table 1:** Patient characteristics and pathologic features of the 768 patients with conventional papillary thyroid carcinoma.

Characteristics	Values
**Age:** n (%)	
<55 years	592 (77.1%)
≥55 years	176 (22.9%)
**Sex:** n (%)	
Female	648 (84.4%)
Male	120 (15.6%)
**Pathological tumor size, mm**: median (range)	16 (2–65)
**Cervical lymph node metastasis (LNM):** n (%)	
Absent	311 (40.5%)
Present	457 (59.5%)
**Gross extrathyroidal extension:** n (%)	
Absent	33 (4.3%)
Present	735 (95.7%)
^**131**^**I dose, mCi**: median (range)	30 (0–200)
**Distant metastasis:** n (%)	
No	757 (98.8%)
Yes	9 (1.2%)

### Construction of the Radiomics Signature

Of the 730 texture features, the top 40 features were selected in the LASSO Cox regression model based on repeated 10-fold cross-validation. These features were used to build the radiomics signature. The Rad-score calculation formula is presented in the Supplementary Information, where the selected features are presented.

### Association of the Radiomics Signature with Disease-free Survival

At univariate analysis, the radiomics signature was associated with disease-free survival (HR = 4.531, 95% CI: 2.909, 7.056 [*P* < 0.001]) (Table 2). Among clinicopathologic variables, a larger pathological tumor size (HR = 1.042, 95% CI: 1.022, 1.063 [*P* < 0.001]), presence of cervical lymph node metastasis (HR = 4.919, 95% CI: 2.611, 9.267 [*P* < 0.001]), distant metastasis (HR = 8.132, 95% CI: 3.286, 20.120 [*P* < 0.001]), gross extrathyroidal extension (HR = 2.253, 95% CI: 1.040, 4.884 [*P* = 0.040]), and a higher dose of radioactive ablation (RAI) (HR = 1.010, 95% CI: 1.006, 1.013 [*P* < 0.001]) was associated with worse disease-free survival.

**Table 2:** Univariate analysis between variables and disease-free survival.

Variables	Hazard Ratio	95% CI	*P* value
**Age**			
<55 years	1		
≥55 years	1.354	0.844, 2.172	0.208
**Sex**			
Female	1		
Male	1.479	0.879, 2.489	0.14
**Pathological tumor size, mm**	1.042	1.022, 1.063	<0.001
**Cervical LNM**			
Absent	1		
Present	4.919	2.611, 9.267	<0.001
**Gross extrathyroidal extension**			
Absent	1		
Present	2.253	1.04, 4.884	0.040
**Distant metastasis**			
No	1		
Yes	8.132	3.286, 20.12	<0.001
^**131**^ **I dose**	1.010	1.006, 1.013	<0.001
**Rad-score**	4.531	2.909, 7.056	<0.001

At multivariate analysis, the radiomics signature was independently associated with disease-free survival (HR = 3.087, 95% CI: 1.931, 4.935 [*P* < 0.001]). Among clinicopathologic variables, the presence of cervical lymph node metastasis (HR = 3.585, 95% CI: 1.849, 6.952 [*P* < 0.001]), distant metastasis (HR = 3.449, 95% CI: 1.329, 8.950 [*P* = 0.011]), and a higher dose of RAI ablation (HR = 1.005, 95% CI: 1.001, 1.009 [*P* = 0.009]) was associated with worse disease-free survival.

### Assessment of the Incremental Value of the Radiomics Signature in DFS prediction

The clinicopathologic model for predicting disease-free survival yielded a C-index of 0.721 (95% CI: 0.675, 0.780). We created a radiomics model that integrated the radiomics signature with all clinicopathologic data, and found that adding the radiomics signature to the clinicopathologic model yielded an improvement of 0.056 (95% CI: 0.023, 0.096) in the C-index, showing improved classification accuracy for disease-free survival (Table 3).

**Table 3:** Performance of the clinicopathologic model and radiomics model.

Variables	Clinicopathologic model			Radiomics model^*^ = Clinicopathologic data + radiomics signature		
	HR	95% CI	P value	HR	95% CI	P value
**Age, years**						
<55	1			1		
≥55	1.669	1.0296, 2.706	0.0377	1.495	0.921, 2.426	0.104
**Sex**						
Female	1			1		
Male	1.205	0.6969, 2.084	0.5043	1.054	0.608, 1.829	0.851
**Pathological tumor size, mm**	1.027	1.0053, 1.049	0.0145	1.012	0.989, 1.036	0.301
**Cervical LNM**						
Absent	1			1		
Present	3.826	1.9727, 7.419	<0.0001	3.585	1.849, 6.952	<0.001
**Gross extrathyroidal extension**						
Absent	1			1		
Present	1.399	0.6248, 3.132	0.4144	1.145	0.497, 2.638	0.750
**Distant metastasis**						
No	1			1		
Yes	5.78	2.2487, 14.858	0.0003	3.449	1.329, 8.950	0.011
**RAI dose**	1.005	1.0016, 1.009	0.0047	1.005	1.001, 1.009	0.009
**Rad-score**				3.087	1.931, 4.935	<0.001
**C-index (95% CI)** ^†^	(0.675, 0.780)			0.777 (0.735, 0.829)		

^*^The radiomics model integrated the radiomics signature (Rad-score) with clinicopathologic data.

^†^Difference between the two c-indexes = 0.777−0.721 = 0.056 (bootstrapped 95% CI: 0.023, 0.096).

## Discussion

In the current study, we evaluated the ability of multi-feature-based radiomics to help estimate disease-free survival in patients with conventional PTC. To our knowledge, this is the first study to apply radiomics in the estimation of prognosis in patients with PTC. The radiomics signature was identified as an independent prognostic factor, and added incremental value to other clinico-pathologic risk factors when estimating individualized disease-free survival. Our study demonstrates the potential of applying a radiomics approach to conventional PTC.

As PTC is generally associated with an excellent long-term mortality, setting disease-free survival from recurrence or persistent disease as the focus endpoint for risk stratification, rather than mortality, would benefit more patients by potentially aiding in individualized treatment and management. In efforts to achieve such risk-stratification, previous researchers have focused on identifying clinico-pathologic risk factors associated with recurrent or persistent disease^19–22^. Yet, these studies generally included various histologic subtypes and used conventional risk factors which are only obtainable after treatment is completed. In our study, we found that the radiomics signature, which is obtained from preoperative images, was independently associated with disease-free survival (HR = 3.087) and could provide more prognostic information prior to the initiation of treatment.

Although radiomics has shown potential in other cancers, research in thyroid cancer has been relatively limited. Previous studies on radiomics in thyroid disease have mostly focused on differentiating benign and malignant nodules or detecting lymph node metastasis by using relatively simple histogram and texture analysis techniques^14–16,18,23^. In our study, for the construction of the radiomics signature, 730 candidate radomics features were reduced to 40 potential predictors through the LASSO cox regression model, which is known as a useful method for feature selection in high-dimensional data^13,24^. Whereas previous staging systems and nomograms, including the American Joint Committee on Cancer (AJC) tumor node metastasis (TNM) staging system, have shown excellent discriminatory ability for mortality prediction with AUC values of 0.89–0.98, the AUC values of nomograms for recurrence prediction in thyroid cancer have been slightly lower, ranging from 0.72–0.76^19,25–29^. In our study, the radiomics signature that combined multiple individual imaging features significantly improved the predictive accuracy of the clinicopathologic model, yielding a C-index of 0.777 (95% CI: 0.735, 0.829). Our study suggests that combining radomics data with other clinicopathologic risk factors may increase the power of decision support models, and aid in achieving personalized estimation of disease-free survival in PTC.

There are some limitations to this study, such as the retrospective nature of its data collection and the relatively small sample size. Another limitation is the lack of external validation and a separate validation data set. Therefore, further studies are needed to overcome these limitations and to validate our results for better generalization. Although the prospective cohort study design would be the preferred study design of such research, the long wait required to analyze survival outcome in PTC, due to its generally excellent prognosis, makes such research daunting to perform. In addition, the small number of events makes it difficult to yield reliable results with smaller data sets. Although we were unable to perform such an independent validation, to our knowledge, this is the first study to apply multi-feature-based radiomics in the estimation of prognosis in patients with PTC. Our results indicate that radiomics has the potential to be a tool for risk stratification, but further validation is needed.

In conclusion, our preliminary study shows that the identified radiomics signature has the potential to be used as a biomarker for risk stratification in patients with conventional PTC. The radiomics model, which incorporated the radiomics signature with clinicopathologic data, showed a significant improvement in discrimination performance for evaluating disease-free survival. Although promising, these are preliminary results and further validation is required on a larger and independent data set before clinical application. After validation, the radiomics signature may serve as a potential tool to guide individualized management for patients with PTC, of whom the majority will have excellent prognosis.

## Methods

### Patients

The institutional review board of Severance hospital approved this retrospective study and the requirement for informed consent was waived. Our institutional database was reviewed to identify patients with histologically confirmed conventional papillary thyroid carcinoma who underwent preoperative US and thyroid surgery from January 2004 to February 2006. In total, 768 patients were identified (648 women and 120 men; median age, 45 years [range, 17–80 years]; tumor size, 16 mm [range, 2–65 mm]) and comprised our study population. Among the study patients, 299 patients were included in a prior study which compared the diagnostic accuracy of preoperative staging using US imaging and CT^30^, and 469 patients were included in a prior study which investigated whether conventional US features were associated with tumor recurrence in PTC^31^.

### Surgery and Follow-up

Total or near-total thyroidectomy was performed in patients who had multiple tumors, extrathyroidal invasion or lymph node metastasis (LNM) on either preoperative or intraoperative findings. Central compartment neck dissection including the paratracheal, pretracheal, and prelaryngeal lymph nodes is routinely performed at our institution. Bilateral central compartment neck dissection was performed in patients who underwent total or near-total thyroidectomy and ipsilateral central compartment neck dissection was performed in patients who underwent hemithyroidectomy. Lateral compartment neck dissection was performed in selected patients with lateral LNM diagnosed by preoperative US-guided fine-needle aspiration. If suspicious LNs were found during surgery, intraoperative frozen biopsy was performed. In patients confirmed to have lateral LNM, lateral neck compartments including levels 2, 3, 4 and anterior level 5 were dissected.

For postoperative follow-up, clinical examination, neck US, chest radiographs, and measurements of serum thyroid-stimulating hormone (TSH), free T4, thyroglobulin (Tg), and anti-Tg antibody were recommended annually. For patients with suspected recurrence chest computed tomography (CT), magnetic resonance imaging (MRI), whole body bone scan or fluorodeoxyglucose positron emission tomography (PET) was performed at the discretion of the physician.

Clinicopathologic data including age, gender, pathological tumor size, cervical lymph node metastasis (LNM), gross extrathyroidal extension, surgery method (total or near-total thyroidectomy, hemithyroidectomy, and hemithyroidectomy with contralateral subtotal thyroidectomy), and radioiodine ablation dose were collected from medical records. Medical records and imaging studies during postoperative surveillance were reviewed for patient outcome.

No evidence of disease was defined as no biochemical (suppressed thyroglobulin (Tg) < 1 ng/mL, stimulated Tg < 2 ng/mL, with negative anti-Tg antibodies) or structural recurrences (no evidence of disease on US, cross-sectional and/or nuclear imaging) during follow-up^32^. Distant metastasis was defined as the development of thyroid cancer foci at distant organs located in areas other than the neck, which was either confirmed with biopsy or clinically suspected based on various imaging studies. Recurrence/persistence of disease was defined by biochemical, structural or functional evidence of disease that was detected with/without a period of any evidence of disease since initial surgery^32^. Disease-free survival was defined as the time of interval (in months) between initial surgery and occurrence of recurrence/persistence or the date of last clinical follow-up.

### US Examinations

All patients underwent preoperative US including both thyroid glands and cervical regions, performed by using a 7–12-MHz (HDI 3000 or 5000; Philips Medical Systems, Bothell,Wash), 5–13-MHz (SONOLINE Antares; Siemens Medical Solutions, Erlangen,Germany/Acuson Sequoia 512; Acuson, Mountain View, CA), or a 5–12-MHz linear array transducer (iU22; Philips Medical Systems, Bothell,Wash).

### Radiomics Feature Analysis

For image feature extraction, a representative US image was selected for each tumor from images that were previously captured by the radiologist at the time of the US examination, and which were retrieved from the picture archiving and communication system. Manual segmentation of the thyroid tumors was performed by a radiologist (V. Y. P) who had 7 years of experience in thyroid US imaging. A region of interest (ROI) was delineated around the boundary of the index tumor on a representative US image, which was validated by a senior radiologist (K.J.Y) who had 16 years of experience in thyroid US imaging.

**Figure 1:**
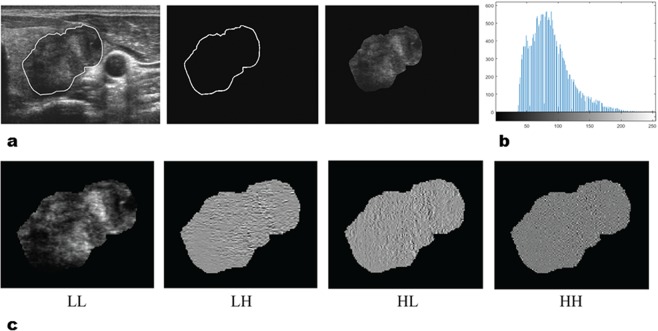
Example of the radiomics feature extraction. (**a**) Each tumor was first manually segmented on a representative US image (left) and subsequently, the position information of the ROI (middle) was collected and applied to the US image without marking the ROI itself, allowing the ROI to be extracted from the original US image (right). (**b**) Intensity histogram of the ROI image is shown. First and second order statistics values were calculated for each image. (**c**) For further feature extraction, the wavelet transform was used. For clearer presentation, the wavelet coefficients were scaled into a range from 0 to 255. From left to right: wavelet decompositions of the original image using LL, LH, HL, and HH, where L and H are low- and high-pass filters in the x- and y-directions, respectively.

The radiomics feature extraction methodology is described in Appendix E1 (online). Texture feature extraction was performed by in-house texture analysis algorithms implemented in MATLAB 2016b (The MathWorks, Inc., Natick, Massachusetts, United States). After saving the ROI-segmented US images as JPG images, the images were then converted into grayscale intensity images by eliminating the hue and saturation information while retaining luminance. A total of 730 candidate radomics features, using GLCM and GLRLM texture matrices, single-level discrete 2D wavelet transform and so forth, were generated from a single US image (Fig. 1). Each image was normalized for direct comparison between patients.

### Statistical Analysis

To select radiomics features, we used the least absolute shrinkage and selection operator (LASSO) method in Cox regression to select the most useful prognostic imaging features for disease-free survival. The LASSO method is a penalized technique for variable selection that is suitable for the regression of high-dimensional data^33^.

In the LASSO Cox regression analysis, 10-fold cross-validation was used to avoid overfitting and to estimate errors of partial likelihood deviance, which is a goodness-of-fit statistic in Cox regression. Additionally, the whole process of partitioning and estimating through cross-validation was repeated 100 times. In each validation step, features were selected by using minimum criteria—i.e., optimal tuning parameter of the LASSO that minimized the partial likelihood deviance was selected and we took the average as the final coefficients for each feature^34^. A larger average coefficient indicates a more relevant feature. Repeated cross-validation can reduce variation caused by the randomness of partitioning the sample into 10-folds as well as estimates of prediction error.

A radiomics score (Rad score) was calculated for each patient as a linear combination of selected features that were weighted by their respective coefficients. The number of features chosen to calculate the radiomics score was determined by the mean number of features selected through 100 repeated cross-validation.

To estimate the association between disease-free survival and radiomics features with clinicopathologic factors, univariable and multivariable Cox proportional hazard regressions were performed. Hazard ratios with 95% confidence interval (CI) for each variable were estimated. To evaluate the incremental prognostic value of the radiomics score when added to clinicopathologic factors, Harrell’s C-index was calculated and compared using the bootstrap method with 1,000 resampling. The significance of the incremental values of the radiomics score was determined using 95% CI for the difference in C-index.

The statistical analysis was performed using R software, version 3.3.3 (http://www.R-project.org). The LASSO method and Cox regression was performed using the “glmnet” and “survival” R packages, respectively. Bootstrapping was implemented using the “boot” R package. P values < 0.05 indicated statistical significance.

## Supplementary information

Supplementary Information
