# Supplementary material for: Association Between Radiomics Signature and Disease-Free Survival in Conventional Papillary Thyroid Carcinoma
Source: Sci Rep. 2019 Mar 14;9:4501. doi: 10.1038/s41598-018-37748-4 (PMC6418281; doi:10.1038/s41598-018-37748-4)
Supplement: Supplementary file 1 — Supplementary Information [file 41598_2018_37748_MOESM1_ESM.docx]

**Title:**

**Association Between Radiomics Signature and Disease-Free Survival in Conventional Papillary Thyroid Carcinoma**

Contributing Authors:

Vivian Y. Park, M.D, PhD.^1^, Kyunghwa Han, PhD^1^, Eunjung Lee, PhD^2^, Eun-Kyung Kim MD, PhD^1^, Hee Jung Moon, MD, PhD^1^, Jung Hyun Yoon, MD, PhD^1^, Jin Young Kwak, M.D., PhD^1*^

Affiliations:

1. Department of Radiology, Severance Hospital, Research Institute of Radiological Science, Yonsei University College of Medicine

2. Department of Computational Science and Engineering, Yonsei University

**Supplementary Methods 1. Radiomics feature extraction methodology**

For feature selection, a total of 768 representative US images were chosen from images that were previously captured by the radiologist at the time of the examination, which were retrieved from the picture archiving and communication system. The ROIs of the thyroid tumors were manually segmented by a radiologist (V.Y.P.) who had 7 years of experience in thyroid US imaging. Once the ROI was delineated around the boundary of the index tumor on the representative US image, the position information of the ROI was collected and applied to the US image without marking the ROI itself, allowing the ROI to be extracted from the original US image.

First, the first order statistics values were collected such as energy, entropy, kurtosis, skewness, standard deviation, variance, maximum, minimum, median, mean, mean absolute deviation, range, root mean squares, uniformity. In order to collect the textural features which describes patterns or the spatial relations with neighborhood pixels, the gray level co-occurrence matrix (GLCM) and gray level run-length matrix (GLRLM) were calculated after each extracted ROI image had been normalized for fair comparison (using min-max). The histogram of intensities of each individual ROI image was calculated using ‘imhist’ function in MATLAB 2016b with 256 bins using a bin width of 1 (Supplementary Figure 1). The GLCM with distance 1 and GLRLM were calculated in angles 0, 45, 90, and 135 degrees. Then corresponding autocorrelation, cluster prominence, cluster shade, cluster tendency, contrast, correlation, difference entropy, dissimilarity, energy, energy (H), homogeneity1, homegeneity 2, informational measure of correlation 1, informational measure of correlation 2, inverse difference moment normalized, inverse difference normalized, inverse variance, maximum probability, sum average, sum entropy, sum variance, variance, short run emphasis, long run emphasis, gray level non-uniformity, run length non-uniformity, run percentage, low gray level run emphasis, high gray level run emphasis, short run low gray level emphasis, short run high gray level emphasis, long run low gray level emphasis, long run high gray level emphasis were obtained.


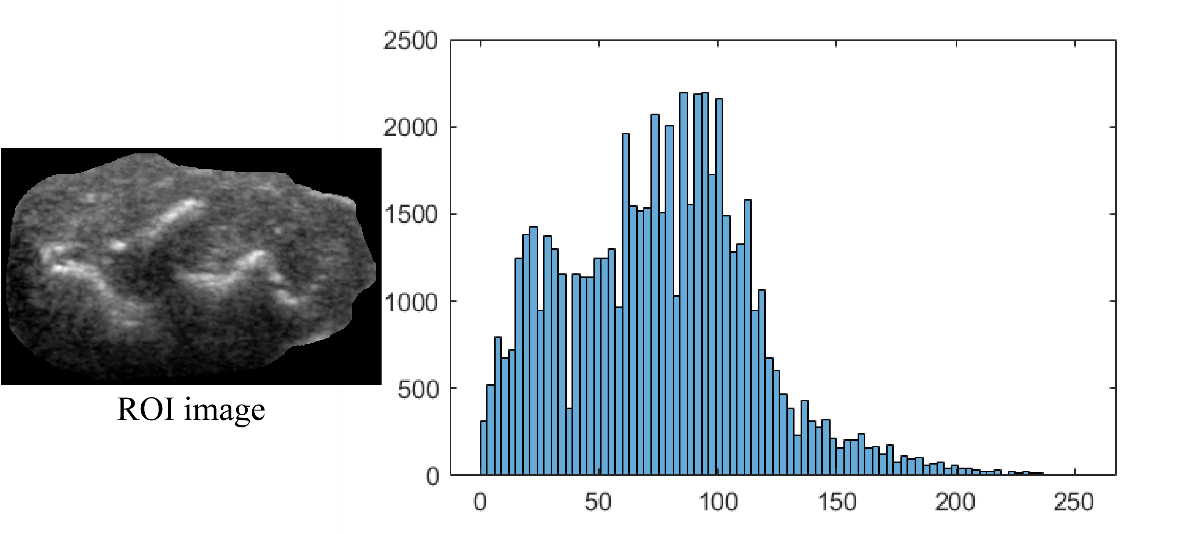


**Supplementary Figure 1.** Histogram of ROI area

*
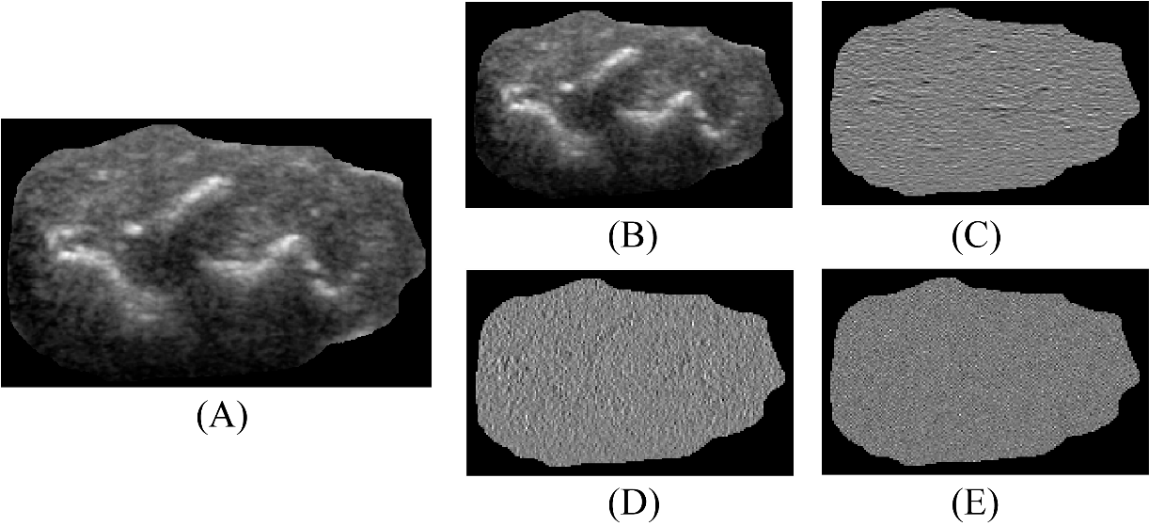
*For further feature extraction, we used the 2 dimensional discrete one-level wavelet transform to decompose the original US image using ‘coiflet 1’ wavelet. The four discrete weavelet decompositions, LL, LH, HL, HH were utilized, where L and H are low- and high-pass filters in the x- and y-directions, respectively. This one-level wavelet decomposition generates four subimages for which LL delineates an approximated image of the original image, LH delineates the horizontal details, HL delineates the vertical details and HH delineates the diagonal details (Supplementary Figure 2). With these four subimages, all the above first order statistics as well as GLCM, GLRLM related feature values were calculated and used in feature analysis.

**Supplementary Figure 2.** Wavelet transformation: (A) original ROI (B) LL (C) LH (D) HL (E) HH

The mathematical formulas for the aforementioned first order statistics values and textural features can be found in the following references and references therein. We will only introduce the formulas for values that are used in the radiomics score calculation in Supplementary Methods 2.

**Reference:**

1. Aerts, H. J. et al. Decoding tumour phenotype by noninvasive imaging using a quantitative radiomics approach. Nat Commun 5, 4006, https://doi.org/10.1038/ncomms5006 (2014).
2. Randen, T., Husoy, J. H. Filtering for texture classification: A comparative study. IEEE Transactions on Pattern Analysis and Machine Intelligence 21, 291-310 (1999).
3. Chang, Y. et al. Computer-aided diagnosis for classifying benign versus malignant thyroid nodules based on ultrasound images: A comparison with radiologist-based assessments. Med Phys 43, 554, https://doi.org/10.1118/1.4939060 (2016).

**Supplementary Methods 2. Radiomics score (Rad-score) calculation formula**

Rad score = uni $\times$ -5.6049 + srlgle_0 $\times$ 4.0807 + LL_imc2_0 $\times$ 3.9196

+ LH_srlgle_90 $\times$ 3.5515 + HH_sre_135 $\times$ 3.0746

+ LH_sre_90 $\times$ 3.0244 + LH_srlgle_135 $\times$ 2.5516

+ LL_srlgle_135 $\times$ 2.4784 + LH_sre_0 $\times$ -2.4154

+ HH_srlgle_135 $\times$ 2.3671 + HH_iv_135 $\times$ -2.2909

+ LH_lglre_90 $\times$ -2.2832 + LH_iv_90 $\times$ 2.102

+ LH_srlgle_45 $\times$ -2.068 + HL_iv_0 $\times$ -1.9875

+ LL_idmn_135 $\times$ -1.9821 + imc2_0 $\times$ 1.7727

+ HL_srlgle_135 $\times$ -1.6802 + LH_srlgle_0 $\times$ 1.5592

+ HL_iv_90 $\times$ 1.5519 + srlgle_135 $\times$ 1.478

+ HH_iv_0 $\times$ -1.3664 + HH_lglre_90 $\times$ -1.3413

+ HH_mad $\times$ 1.0349 + HH_imc1_45 $\times$ -0.8362

+ HH_imc2_0 $\times$ -0.783 + LL_imc2_45 $\times$ -0.7466

+ LL_se_45 $\times$ 0.7465 + LH_de_90 $\times$ -0.739

+ LL_rp_45 $\times$ -0.7294 + LH_iv_45 $\times$ 0.7252

+ LL_imc1_90 $\times$ -0.6999 + LL_rp_135 $\times$ -0.6794

+ HL_sre_135 $\times$ -0.6691 + LH_imc2_0 $\times$ 0.6512

+ HH_med $\times$ -0.6472 + LL_rp_0 $\times$ -0.6302

+ HL_imc1_45 $\times$ 0.627 + HH_sre_90 $\times$ 0.5966

+ imc1_0 $\times$ -0.507

where the values initiating with LL, LH, HL and HH are the quantities obtained from each wavelet subimages, the values without them are from the original image, and the numbers 0, 45, 90, 135 indicates the directions when GLCM and GLRLM are calculated. The abbreviations in the above ‘Rad score’ are collected as follows.

First order statistics: let **J** be the image matrix with *n* pixels and and let **P** be the first order histogram with 256 intensity levels. Then ‘mad=mean absolute deviation’, ‘med=median’ and ‘uni=uniformity’ are calculated as

$$\text{mad=}\frac{1}{n}\sum_{k=1}^{n} \left| \mathbf{J}\left( k \right)-\mathrm{mean}\left( \mathbf{J} \right) \right|, med=median of \mathbf{J,}\text{uni=}\sum_{k=1}^{256} \mathbf{P}\left( k \right)^{2}\mathbf{.}$$

Gray level co-occurrence matrix (GLCM): let **M** be the gray level co-occurrence matrix with adjacent to one pixel apart in the direction 0, 45, 90 or 135. The number of levels is set to 10, that is the values of **J** are rescaled as integers between 1 to 10, based on the minimum and maximum values of given image intensity. Then we have ‘de=difference entropy’, ‘idmn=inverse difference moment normalized’, ‘imc1=informational measure of correlation 1’, ‘imc2=informational measure of correlation 2’, ‘iv=inverse variance’ and ‘se=sum entropy’ with ‘ent(f)=the entropy of f’, $de=\sum_{k=0}^{9} m_{m}\left( k \right) \log_{2} m_{m}\left( k \right)$,

$$idmn=\sum_{i=1}^{10} \sum_{j=1}^{10} \frac{\mathbf{M}\left( i,j \right)}{1+\frac{\left( i-j \right)^{2}}{100}}, iv=\sum_{i=1}^{10} \sum_{j=1}^{10} \frac{\mathbf{M}\left( i,j \right)}{\left( i-j \right)^{2}}\left( i\neq j \right), imc1=\frac{\alpha-a1}{\max\{e\left( m_{x} \right),e\left( m_{y} \right)\}}, imc2=\sqrt{1-e^{-2(a2-\alpha)}}, se=-\sum_{k=2}^{20} m_{p}\left( k \right)\log_{2} m_{p}(k)$$

in which $m_{x}\left( k \right)=\sum_{j=1}^{10} \mathbf{M}(i,j),m_{y}\left( k \right)=\sum_{i=1}^{10} \mathbf{M}(i,j), \alpha=-\sum_{i,j=1}^{10} \mathbf{M}\left( i,j \right)\log_{2} \mathbf{M}\left( i,j \right)$,

$$m_{p}\left( k \right)=\sum_{i=1}^{10} \sum_{j=1}^{10} \mathbf{M}(i,j), \begin{matrix} i+j=k \\ k=1,\cdots,20 \end{matrix} , m_{m}\left( k \right)=\sum_{i=1}^{10} \sum_{j=1}^{10} \mathbf{M}(i,j),\begin{matrix} |i-j|=k \\ k=0,\cdots,9 \end{matrix} ,$$

and $a1=-\sum_{i,j=1}^{10} \mathbf{M}\left( i,j \right)\log m_{x}\left( i \right)m_{y}\left( j \right), a2=-\sum_{i,j=1}^{10} m_{x}\left( i \right)m_{y}(j)\log m_{x}\left( i \right)m_{y}\left( j \right)$.

Gray level run-length matrix (GLRLM)

: let **G** be the gray level run-length matrix and it provides the size of homogeneous runs for each gray level (we set 16 gray levels) in the direction of 0, 45, 90, and 135. The parameters are ‘lglre = low gray level run emphasis’, ‘rp= run percentage’, ‘sre= short run emphasis’ and

‘srlgle = short run low gray level emphasis’ that are calculated as $rp=\sum_{i=1}^{16} \sum_{j=1}^{N} \mathbf{G}(i,j)/n,$

$$lglre=\frac{\sum_{i=1}^{16} \sum_{j=1}^{N} \mathbf{G}(i,j)/i^{2}}{\sum_{i=1}^{16} \sum_{j=1}^{N} \mathbf{G}(i,j)},sre=\frac{\sum_{i=1}^{16} \sum_{j=1}^{N} \mathbf{G}(i,j)/j^{2}}{\sum_{i=1}^{16} \sum_{j=1}^{N} \mathbf{G}(i,j)},\mathrm{srlgle}=\frac{\sum_{i=1}^{16} \sum_{j=1}^{N} \mathbf{G}(i,j)/{(i}^{2}j^{2})}{\sum_{i=1}^{16} \sum_{j=1}^{N} \mathbf{G}(i,j)}$$

where N =max(r,c) with rxc-quantized matrix of given image (or image matrix **M**) using quantization level 16.
